# Reagent-free phosphorus precipitation from a denitrified swine effluent in a batch electrochemical system

**DOI:** 10.1016/j.heliyon.2024.e36766

**Published:** 2024-08-23

**Authors:** Emma Dessì, Emma Company, Narcís Pous, Stefano Milia, Jesús Colprim, Albert Magrí

**Affiliations:** a Laboratory of Chemical and Environmental Engineering (LEQUIA), Institute of the Environment, University of Girona, Girona, Spain; b University of Cagliari, Department of Civil-Environmental Engineering and Architecture (DICAAR), Cagliari, Italy; c National Research Council, Institute of Environmental Geology and Geoengineering (CNR-IGAG), Cagliari, Italy

**Keywords:** Electrochemical mediated precipitation, Phosphorus recovery, Water electrolysis, Current density, Denitrified swine effluent, Precipitated phosphate salts

## Abstract

There is high interest in the recovery of phosphorus (P) from wastewater through crystallization processes. However, the addition of chemical reagents (e.g., sodium hydroxide) to raise the pH may result in high treatment costs and increased concentrations of undesired metal ions (e.g., sodium). As an alternative, in this research we considered electrochemical mediated precipitation at low current densities (0.4–1.2 A m^−2^) without using chemical reagents. For that purpose, a two-chamber electrochemical system was operated in batch for treating denitrified swine effluent (48 mg P L^−1^). By applying current at 1.2 A m^−2^, and targeting pH 11.5, a maximum P removal rate of 33.4 mmol P (L·d^−1^) was obtained while the P removal efficiency was above 90 %. New solids that formed mostly remained suspended in the catholyte. Before discharge, the catholyte effluent was recirculated to the anodic compartment to neutralize the pH, achieving a final pH of 6.4 ± 0.1. Chlorine (Cl_2_) production in the anodic compartment was favored by a small anode surface and a high initial pH of the catholyte. Although the production of chlorine achieved was limited (the highest concentration was 8.6 ± 0.1 mg Cl_2_ L^−1^) these findings represent a new opportunity for the recovery and onsite use of this side-product. Electrochemical impedance spectroscopy tests confirmed that the deposition of solids inside the cathodic compartment during the experimental period was limited. Membrane analysis revealed significant scaling of carbonate compounds. The electrochemical treatment described above was shown as a promising alternative to sodium hydroxide and sulfuric acid dosage for pH adjustment when crystallizing phosphate salts.

## Highlights

• Denitrified swine effluent was electrochemically treated in a batch system.
• Chemical-free pH adjustment allowed PO_4_^3−^ crystallization and effluent neutralization.
• Low current density (≤1.2 A m^−2^) induced MgP formation in the bulk liquid.
• EIS confirmed the limited deposition of solids into the system.
• Chlorine production in the anodic chamber was favored by a small anode surface.

## Graphical abstract

**Figure:**
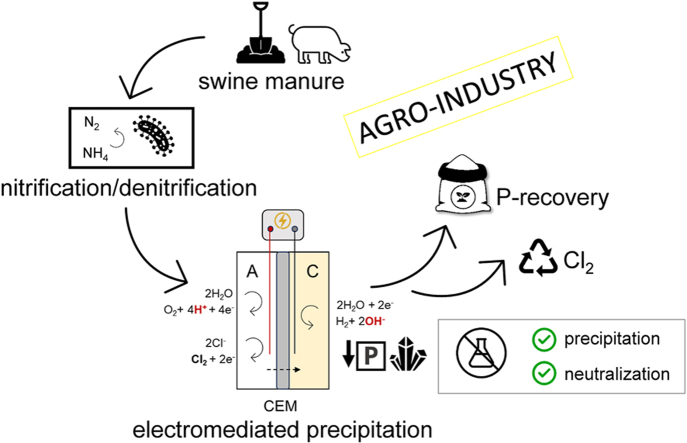

## Abbreviations:

ADP: adenosine diphosphate
ALK: alkalinity
AMP: adenosine monophosphate
ATP: adenosine triphosphate
CaP: calcium phosphate
CD: current density
CEM: cation exchange membrane
DPD: N,N-diethyl-p-phenylenediamine;
DNA: deoxyribonucleic acid
EC: electrical conductivity
ECM: electrical circuit model
EDTA: ethylenediaminetetraacetic acid
EIS: electrochemical impedance spectroscopy
EMP: electrochemical mediated precipitation
ET: electrochemical technology
IAP: ion activity product
ICP-OES: inductively coupled plasma-optical emission spectrometry
IRE: ion removal efficiency
MgP: magnesium phosphate
MMO: mixed metal oxide;
OCV: open circuit voltage
P: phosphorus
pHRR: pH raising rate
PRE: P removal efficiency
PRR: P removal rate
RNA: ribonucleic acid
SD: standard deviation
sEC: specific energy consumption
sEC_P_: sEC referred to P-removal
SEM: scanning electron microscope
SI: saturation index
TIC: total inorganic carbon
XRD: X-ray diffraction

## 1 Introduction

Phosphorus (P) is an essential element for all living organisms as a constituent of, among others, cell membranes, nucleic acids (DNA and RNA) and energy-transfer molecules in metabolism (ATP, ADP, and AMP) [1]. The shortage of this irreplaceable nutrient limits food production, but when discharged in excess to the environment it acts as a pollutant, causing nutrient imbalances and eutrophication [2]. The mining industry typically extracts P from phosphate rock, a non-renewable resource. As reserves of phosphate rock available in geological deposits unevenly distributed worldwide are being depleted, uncertainties arise in the supply of this nutrient [3,4]. Alternatively, P can be recovered from wastewater streams and livestock effluents, a renewable source of nutrients available at the local scale, using multiple methods [[5], [6], [7]] such as the crystallization of low-soluble phosphate salts (e.g., hydroxyapatite, struvite, vivianite, etc.) [[6], [8], [9]], which could be used directly in agriculture.

The pH is related to the induction of supersaturation conditions [10,11] so this is a key factor in the crystallization reactions. The addition of chemical reagents is traditionally used to raise the pH value (e.g., sodium hydroxide (NaOH)) besides increasing the content in metal ions (e.g., calcium hydroxide (Ca(OH)_2_), magnesium oxide (MgO), etc.). Commonly, once P has been recovered, the pH of the liquid stream needs to be neutralized again by the addition of chemical reagents (e.g., sulfuric acid (H_2_SO_4_)). An alternative method to modulate the pH without the use of chemical reagents can be found in electrochemistry.

Water electrolysis has been applied in water treatment as a clean method for the onsite production of hydroxyl ions (OH^−^) [[12], [13], [14]]. An electrochemical system typically consists of electrodes (i.e., anode and cathode), an electrolyte solution, and an external power supply. When current is supplied, water molecules at the cathode are reduced to hydrogen (H_2_) with the simultaneous production of OH^−^ (Reaction (1)), which raises the local pH, while at the anode, water molecules are oxidized to oxygen (O_2_) and protons (H^+^) (Reaction (2)), neutralizing the OH^−^ produced at the cathode. Ion exchange membranes are used in electrolysis cells to prevent the neutralization of the low-pH anolyte with the high-pH catholyte. In this case, the system is typically split into two compartments and a pH-gradient is formed between the anodic and cathodic compartments. Furthermore, side-reactions such as chloride (Cl^−^) conversion to chlorine gas (Cl_2_) (Reaction (3)) can take place in the anode, thus leading to the production of other useful compounds:

(1) $$Cathode:{2H}_{2}O+{2e}^{-}\to H_{2}+{2OH}^{-}$$

(2) $$Anode:{2H}_{2}O\to O_{2}+{4H}^{+}+{4e}^{-}$$

(3) $${2Cl}^{-}\to {Cl}_{2}+{2e}^{-}$$

In recent years, wastewater treatment has experienced a growing interest in electrochemical technologies (ETs), including P removal and recovery applications [15,16]. The interest in neutralizing the pH of the wastewater by electrochemical means has also been referred to earlier [17,18]. The ETs usually are compact, allowing the production of chemical reagents in situ, thus avoiding their transportation and storage. The electrochemical mediated precipitation (EMP) process is advantageous with respect to other ETs, such as electrochemical coagulation. The latter process applies sacrificial electrodes to release cations (e.g., aluminum, iron, or magnesium) to remove phosphate, which will produce a large amount of sludge, and the electrodes need to be replaced regularly during long-term operation [19], leading to an increase in the operational costs. By contrast, the EMP process will produce P minerals and can employ inert electrodes, which are not consumed during the reaction [16]. The EMP process has already been applied in several situations for the recovery of precipitated phosphate salts. This is the case, for instance, of calcium phosphate from urban wastewater [20] and cheese wastewater [14], ferric phosphate from hypophosphite-laden wastewater [21], and struvite from digestate [22].

Multiple factors affect the performance of an EMP process, such as the pH, water matrix, current density (CD), and cell configuration [16], which need to be evaluated to minimize the energy consumption of the process. The electrodes are usually inert and the removal of P from the liquid phase is driven by the production of OH^−^ and the achievement of high pH values. The energy consumption, nonetheless, may be high when targeting fast P removal rates [23,24]. When no membrane is used in the electrochemical system, cations available in the wastewater are headed toward the cathode due to electro-migration, and most of the precipitate becomes attached to the cathode's surface (due to the local high pH), from where it needs to be collected periodically [20]. This is because excessive coating on the electrode leads to an increase in ohmic resistance eventually forcing cathode deactivation [25]. The need to pause the treatment to collect the precipitate is limiting for the long-term operational feasibility. Beyond manual cleaning, polarity inversion has also been proposed as a method for detaching precipitates [26]. Alternatively, the anode and the cathode can be separated by a cation exchange membrane (CEM), allowing for a differentiated pH in both compartments. The high pH in the cathode compartment causes immediate precipitation of the phosphate in the liquid and, consequently, precipitation at the electrode surface hardly takes place. After crystallization, a final separation by centrifugation, settling or filtration is necessary for the final recovery of the phosphate compounds [27]. To contribute to the development of an energy-efficient electrochemical process while limiting the precipitation on the electrode surface, the application of a low CD could be a viable option [28]. Under these conditions, the co-precipitation of calcium carbonate (CaCO_3_) and brucite (Mg(OH)_2_) was also reduced.

In the present work, denitrified swine effluent was used to explore the feasibility of reagent-free P recovery through an EMP process. Considering the previous experience reported by Company et al. [29] with this kind of effluent based on the dosage of chemical reagents (i.e., NaOH) to raise the pH to values as high as 10.5–11.5 and H_2_SO_4_ to decrease the pH to around 7.0, EMP at low CD (≤1.2 A m^−2^) was tested as an alternative. This new approach, with no other similar cases reported in the field of livestock effluents, also led to an arrest in the supply of Na^+^ to the system, which can affect the quality of the recovered product. Particularly, the influence of the targeted pH, wastewater strength and CD on P removal from the liquid phase was investigated using a two-chamber electrochemical system equipped with a CEM and running in batch mode. Thus, precipitation tests were performed to assess the potential for P removal in the cathodic compartment and the energy consumption efficiency of the system. The recovered products were characterized. Moreover, catholyte neutralization before discharge was also explored in the anodic compartment in view of operating the system in continuous-flow mode. Electrochemical impedance spectroscopy (EIS) was used as a fast and non-destructive technique to study the performance of the electrochemical system and, particularly, the resistance increase due to precipitation phenomena.

## 2 Material and methods

### 2.1 Batch electrochemical system setup

**Fig. 1:**
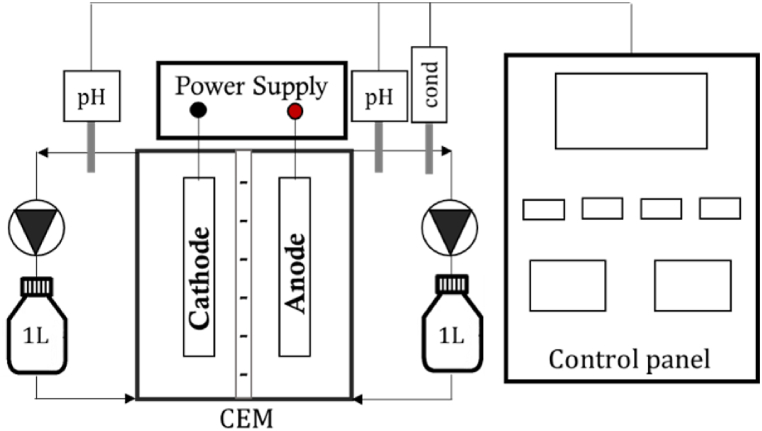
Scheme of the batch electrochemical system setup.

A double-compartment square reactor made of methacrylate was used as an electrochemical system (Fig. 1). The anode (frame size: 28 x 28 × 2 cm^3^, working volume: 830 mL) and the cathode (frame size: 28 x 28 × 3 cm^3^, working volume: 1010 mL) compartments were separated by a CEM (CMI-7000, Membranes Int., USA) with a surface of 784 cm^2^. A stainless-steel mesh with a total surface of 2418 cm^2^ (1.0 mm of light path and 0.4 mm wire diameter, CISA, Spain) was used as the cathode, and a Ti-MMO mesh with a surface of 148 cm^2^ (2 mm light path and 1 mm wire diameter, NMT electrodes, South Africa) was used as the anode. The reactor was galvanostatically controlled, at a fixed CD value (from 0.4 to 1.2 A m^−2^), using an external power supply (mod. IMHY3003D, Lendher, Spain). Peristaltic pumps (mod. 323, Watson Marlow, UK) were used to recirculate the denitrified effluent (working flow rate: 192 mL/min) in each compartment, both connected to a 1-L Schott bottle. The pH of the catholyte and the pH and electrical conductivity (EC) of the anolyte were recorded online using a control panel that included a multimeter (mod. MM44, Crison Instruments SA, Spain) plugged to a memograph (mod. RSG40, Endress + Hauser Inc., Switzerland).

### 2.2 Denitrified swine effluent characterization

The denitrified swine effluent used in the electrochemical tests was collected from a pig farm located in Osona (Catalonia, Spain). Following solid-liquid separation, the liquid fraction of the slurry is treated biologically onsite, in a sequencing batch reactor under intermittent aeration, aiming to remove nitrogen (N). The denitrified effluent was sampled after the sludge had settled. Samples were transported from the farm to the laboratory in polyethylene containers. Once at the laboratory facilities, these containers were stored at room temperature until performing the experiments. The final compositional characteristics of the denitrified effluent used are given in Table 1. Low alkalinity (1056 mg CaCO_3_ L^−1^) and no ammonium-N remained in the effluent after biological treatment. The Mg^2+^:PO_4_^3−^ molar ratio was 2.8, much higher than the Ca^2+^:PO_4_^3−^ molar ratio, which was 0.7.

**Table 1:** Main physicochemical characteristics of the denitrified swine effluent.

Parameters	Units	Average	SD
pH	–	8.5	0.2
EC	mS cm^−1^	6.1	0.1
ALK (CaCO_3_)	mg L^−1^	1056	26
TIC	mg L^−1^	248	6
Na^+^	mg L^−1^	456	31
K^+^	mg L^−1^	1068	76
Mg^2+^	mg L^−1^	104	8
Ca^2+^	mg L^−1^	41	3
Cl^−^	mg L^−1^	864	40
SO_4_^2-^-S	mg L^−1^	124	7
PO_4_^3-^-P	mg L^−1^	48	5
NH_4_^+^-N	mg L^−1^	0	0
NO_2_^−^-N + NO_3_^−^-N	mg L^−1^	0	0

ALK, alkalinity; EC, electrical conductivity; SD, standard deviation; TIC, total inorganic carbon.

### 2.3 Experimental tests performed in the electrochemical system

#### 2.3.1 Batch experiments

##### 2.3.1.1 Precipitation in the cathodic compartment

Precipitation tests using denitrified swine effluent were performed by operating the electrochemical system in batches (in triplicates). The target was to assess the influence of the operational conditions applied including the final pH value (10.5 and 11.5), CD (0.4, 0.6, 0.8 and 1.2 A m^−2^), and effluent strength (dilution factor with deionized water: 1x -undiluted (1:1)- and 4x (1:4)) on the ion removal rate and efficiency. All tests were run at room temperature (20 ± 2 °C) using ca. 1.65 L of denitrified effluent in each compartment. Depending on the applied current and the effluent strength, the resulting cell potential ranged from 2.4 to 5.0 V. The experiments ended when the pH value reached 11.5. Once at that point, the power supply was switched off and the cathodic and anodic bottles were emptied. The reactor compartments were then cleaned with an acid solution and deionized water to ensure that no residual precipitates remained. The final catholyte was filtered through a filtering paper to retain solids, which were dried at room temperature and ground before analysis. Liquid samples from the anodic and cathodic compartments were collected when the pH of the catholyte reached values of 10.5 and 11.5. These samples were filtered at 0.2 μm and stored at room temperature before analysis. At the end of the experiments, the electrochemical cell was opened to characterize the solids deposited on the surface of the membrane.

##### 2.3.1.2 Neutralization in the anodic compartment

Neutralization tests were carried out to explore whether the catholyte could be potentially neutralized in the anodic compartment before discharge. The solution to be recirculated in the anodic compartment was obtained from the previous precipitation test using undiluted effluent (i.e., final catholyte at pH 11.5), while in the cathodic compartment the recirculated liquid was fresh denitrified effluent. Additionally, the influence of the anode surface on the chlorine production was assessed by tripling the electrode area (i.e., from 148 to 444 cm^2^). Acid requirements for the neutralization (pH 7.0) of the catholyte were assessed titrimetrically using sulfuric acid [29].

#### 2.3.2 Electrochemical impedance spectroscopy (EIS) tests

A BioLogic potentiostat (mod. VSP, France) was used to perform the EIS tests on the batch electrochemical system. A volume of ca. 1.65 L of fresh denitrified effluent was recirculated in each compartment. At least two EIS runs were performed to characterize the system's electrochemical performance prior to and after conducting the precipitation tests. The internal resistance was investigated using two- and three-electrode configurations. In the latest case, an Ag/AgCl sat. KCl reference electrode (+0.197 V vs. SHE, SE 11, Xylem Analytics Germany Sales GmbH & Co. KG Sensortechnik Meinsberg, Germany) was placed in the cathodic compartment, as close as possible to the electrode. Firstly, before any EIS measurement, steady-state conditions were obtained considering voltage stabilization for a minimum of 1.5 h in open circuit voltage (OCV). To ensure that the relevant physical phenomena were captured in the EIS spectrum, all EIS measurements occurred over the frequency range from 100 kHz to 10 MHz. A sinusoidal perturbation with an amplitude of 10 mA was used with 10 points per logarithmic decade for the analysis [30]. A potential of 0 V vs. three different fixed potential values (−0.8, −1.0 and −1.2 V vs. Ag/AgCl) were applied as input signals to investigate the influence on the components of the overall system internal resistance. To study the impedance results, the Nyquist plot was used. In this plot, every interface can ideally be visualized as a semicircle. The EIS parameters were estimated by fitting an equivalent electrical circuit model (ECM) using Zfit (EC-lab software, BioLogic). The ECM produces pseudo-electrochemical parameters, which can be sorted to represent the anode and cathode impedances separately, as well as individually to assess the ohmic, kinetic, and mass transfer limitations of the system. Common configurations of ECM have included a resistor representing solution resistance connected in series to parallel combinations of resistors representing charge transfer reactions, but when the mass transfer is expected to be a limiting factor in system performance Warburg elements are also included [31]. Thus, these above-mentioned elements were used in the fitting model to represent the obtained results. Particularly, the EIS spectra intersection with the X-axis identifies the ohmic resistance of the system.

### 2.4 Analytical methods

Water samples were mostly analyzed following APHA et al. [32]. The pH was measured offline using a bench pH-meter (mod. Sension + PH3, Hach, Germany). Electrical conductivity (EC) measurements were carried out using a conductivity-meter (mod. EC-Meter Basic 30+, Crison Instruments SA, Spain). Total alkalinity (ALK, reported as CaCO_3_) was measured by acid titration to an endpoint pH of 4.5 and total inorganic carbon (TIC; H_2_CO_3_* + HCO_3_^−^ + CO_3_^2−^) was determined through the 5 pH point titration method [33]. The concentration of the soluble cations (i.e., sodium (Na^+^), ammonium (NH_4_^+^), potassium (K^+^), calcium (Ca^2+^), and magnesium (Mg^2+^)), as well as the concentration of the soluble anions (i.e., chloride (Cl^−^), nitrite (NO_2_^−^), nitrate (NO_3_^−^), phosphate (PO_4_^3−^), and sulfate (SO_4_^2−^)), was determined by ion chromatography (mod. ICS-5000, Dionex, USA) after filtering samples with 0.2-μm nylon filters. Chlorine gas (Cl_2_) was measured using a spectrophotometer (mod. DR1900, Hach Lange, Germany) according to the DPD-free chlorine method (Hach Lange). For solid samples, the total content of the elements Na, K, Ca, Mg, and P was measured after microwave digestion with an HNO_3_/H_2_O_2_ mixture using inductively coupled plasma-optical emission spectrometry (ICP-OES) (mod. 5100, Agilent Technologies, USA). The solids formed were also analyzed using X-ray diffraction (XRD) (mod. D8 Advance, Bruker, USA). Membrane-related images were obtained through scanning electrode microscopy (SEM) (mod. DSM-960A, Zeiss, Germany).

### 2.5 Calculations

The hydroxyl production (OH^−^_prod_) [mol OH^−^ L^−1^], was calculated considering the electric current that was applied during the test, and assuming 100 % faradaic efficiency, as shown in Eq. (4) (adapted from Ref. [34]):

(4) $${OH}_{prod}^{-}=\frac{I\cdot \;t_{pH}}{F\cdot V_{CAT}}$$

where: *t*_*pH*_ [s] is the time needed to raise the initial pH of the denitrified effluent in the cathodic compartment up to a given pH value according to the applied current (*I* [A]), *F* is the Faraday constant (96485.332 C mol^−1^) and *V*_*CAT*_ [L] is the recirculating catholyte volume. For comparison purposes, the hydroxyl demand of the denitrified effluent was estimated experimentally based on a titration test using NaOH [29]. The pH raising rate in the cathodic compartment (*pHRR*_*CAT*_) [upH h^−1^] was calculated according to the pH-time slope. To ensure a linear profile, the slope was obtained by linear regression considering the pH values measured between 10.5 and 11.5. The specific energy consumption (*sEC*) [kWh m^−3^] in the electrochemical system was calculated according to Eq. (5) (adapted from Refs. [35,36]):

(5) $$\mathrm{sEC}=\frac{I\cdot \int Vdt}{V_{CAT}}$$

where: *V* [V] is the cell voltage, and *t* [h] is the time the experiment lasted. The P removal rate (*PRR*) [mol (L·d)^−1^] was calculated according to Eq. (6):

(6) $$PRR=\frac{\left(P_{CAT,t0}-P_{CAT,t}\right)}{t_{pH}}$$

where: *P*_*CAT*_ is the molar concentration of P [M] measured in the catholyte at time *t*_*0*_ (i.e., at the start of the test) and time *t* (i.e., at the time when pH 10.5 or 11.5 was reached). Otherwise, the ion removal efficiency (*IRE*) [%], which was referred to PO_4_^3−^, Ca^2+^ and Mg^2+^, was calculated as shown in Eq. (7) [29]:

(7) $$IRE=\frac{\left(C_{CAT,t0}-C_{CAT,t}\right)}{C_{CAT,t0}}$$

where: *C*_*CAT*_ is the ion molar concentration [M] measured in the catholyte at time *t*_*0*_ (i.e., at the start of the test) and time *t* (i.e., at the time when pH 10.5 or 11.5 was reached). The specific energy consumption in relation to the P removal from the liquid phase (*sEC*_*P*_) [kWh kg^−1^ P] is calculated according to Eq. (8), once accounted for the molecular weight of P (*MW*_*P*_) (adapted from Refs. [35,36]):

(8) $${\mathrm{sEC}}_{P}=\frac{\mathrm{sEC}}{\left(P_{CAT,t0}-P_{CAT,t}\right)\cdot {MW}_{P}}$$

The cost of dosing sodium hydroxide was calculated according to 0.50 € kg^−1^ NaOH [12] and considering the result of the titration test for the denitrified effluent (22.8 mmol L^−1^ to reach pH 11.5). The cost of dosing sulfuric acid was calculated according to 0.25 € kg^−1^ H_2_SO_4_ [37] and considering the result of the titration test for the catholyte to decrease the pH from 11.5 to 7.0 (20.8 mmol H^+^ L^−1^). On the other hand, the cost of operating the electrochemical system (i.e., external power supply) was calculated considering the cost of the energy for an industrial application (0.20 € kWh^−1^; source: European electricity price for the second semester of 2022) and the experimental sEC (kWh m^−3^).

Considering the composition of the swine denitrified effluent, the supersaturation conditions and possible mineral phases formed were assessed using the freeware Visual MINTEQ [38]. The saturation index (SI, log_10_(IAP/K_sp_)) was calculated as a function of the corresponding ion activity product (IAP) and the mineral phase solubility product constant (K_sp_). If the SI for a particular mineral is positive, the system is supersaturated with respect to that mineral, and precipitation may occur. Values for the K_sp_ of the mineral phases bobierrite (10^−25.2^), cattiite (10^−23.1^), K-struvite (10^−12.2^), and Na-struvite (10^−11.6^) were added to the Visual MINTEQ original database according to other sources [39,40].

## 3 Results and discussion

### 3.1 Precipitation tests in the cathodic compartment

#### 3.1.1 Influence of current density and denitrified effluent strength

**Fig. 2:**
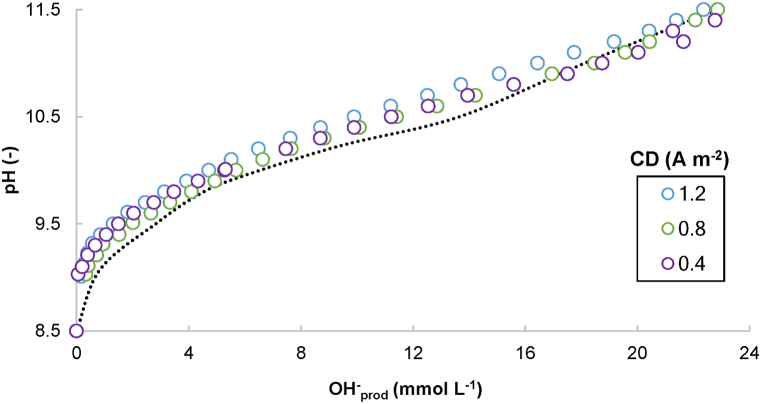
Evolution of the pH in the catholyte as a function of the OH^−^ produced (calculated as a function of time), considering different current densities (CDs). The dashed black line corresponds to the evolution of the pH of the undiluted denitrified effluent by the addition of NaOH.

A two-chamber electrochemical system equipped with a CEM was run in batch mode to precipitate P from a denitrified swine effluent without dosing chemical reagents. The pH of the catholyte increased as a function of the OH^−^ produced (Fig. 2). The pH profiles matched well with the titration curve obtained for the fresh denitrified effluent using NaOH, proving that the estimation performed according to Eq. (4) was close to reality. These results suggest a limited diffusion of OH^−^ and H^+^ through the membrane, which leads to high faradaic efficiency in terms of CD conversion into OH^−^.

**Fig. 3:**
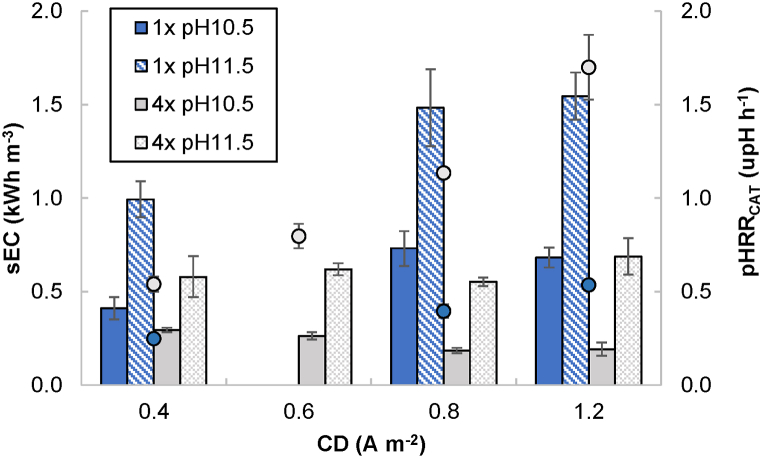
Specific energy consumption (sEC) (bars), and pH raising rate in the cathodic compartment (pHRR_CAT_) (dots), against current density (CD) for 1x and 4x dilution (final pH: 10.5 and 11.5).

All the time-dependent pH profiles registered in the cathodic compartment showed a characteristic pH increase in two stages (Fig. S1) due to the buffering characteristics of the denitrified effluent. The slope at which the pH increased was steeper in the range 8.5–9.5 than in the range 10.5–11.5. For this reason, the pHRR_CAT_ was calculated considering only the second slope in the pH profile (Fig. 3). The pHRR_CAT_ increased according to the CD applied (i.e., the higher the CD the shorter the time needed to reach pH 11.5) but decreased according to the denitrified effluent strength (i.e., the lower the strength -less buffer capacity-, the shorter the time needed). As an example, the time needed by the undiluted effluent to reach pH 10.5 in the cathodic compartment was 2.84 ± 0.45 h and the time needed to reach pH 11.5 was 6.87 ± 0.77 h at 0.4 A m^−2^ (pHRR_CAT_: 0.25 upH h^−1^). This time shortened to 1.43 ± 0.03 and 3.24 ± 0.05 h at 1.2 A m^−2^ (pHRR_CAT_: 0.54 upH h^−1^). If considering 4x diluted effluent the time needed to reach pH 11.5 at 1.2 A m^−2^ was only 0.75 ± 0.08 h (pHRR_CAT_: 1.70 upH h^−1^).

The theoretical cell potential can be calculated with the Nernst equation [41] as a function of the pH reached in both compartments. Thus, a theoretical cell potential of 1.4–1.5 V (pH_CAT_ 10.5) and 1.6–1.7 V (pH_CAT_ 11.5) was eventually required to drive the water reduction reaction in the cathodic compartment and the water oxidation reaction in the anodic compartment. However, due to the existence of overpotentials, ohmic resistance and precipitation phenomena, the cell voltage actually supplied increased during the tests up to 2.4–5.0 V. Thus, real sEC values for undiluted effluent and pH 11.5 reached 0.99 ± 0.10 and 1.55 ± 0.13 kWh m^−3^ when working at 0.4 and 1.2 A m^−2^, respectively. If working with 4x diluted effluent the sEC was calculated as 0.55–0.69 kWh m^−3^. Under the experimental conditions applied, the sEC remained approximately constant for 1x dilution when the CD increased from 0.8 to 1.2 A m^−2^. A similar pattern can be observed in 4x dilution experiments regardless of the CD applied (Fig. 3).

**Fig. 4:**
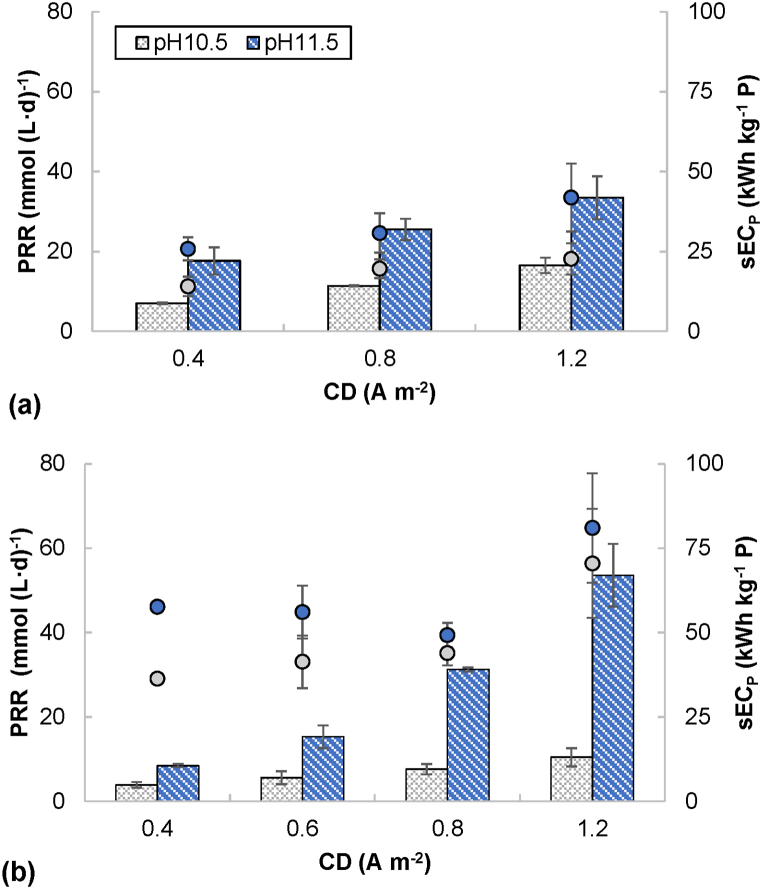
Phosphorus removal rate (PRR) (bars) and specific energy consumption based on P removal (sEC_P_) (dots) against current density (CD) considering (a) 1x and (b) 4x dilution of the denitrified effluent (final pH: 10.5 and 11.5).

**Fig. 5:**
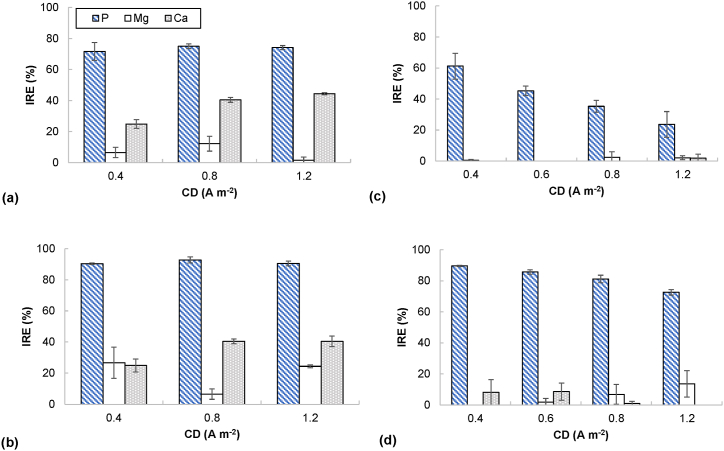
Ions removal efficiency (IRE) against current density (CD) for (a) pH 10.5 and 1x diluted, (b) pH 11.5 and 1x diluted, (c) pH 10.5 and 4x diluted, and (d) pH 11.5 and 4x diluted.

As far as the OH^−^ production rate increases with the current applied, P removal from the liquid phase occurs faster. Thus, the maximum PRR was achieved at the highest CD tested (1.2 A m^−2^) when targeting pH 11.5 (i.e., 33.4 ± 5.3 mmol P (L·d)^−1^ for ×1 dilution and 53.6 ± 7.5 mmol P (L·d)^−1^ for 4x dilution) (Fig. 4a and b). For those experiments concerning no wastewater dilution, the applied CD did not significantly affect the PRE, but that was not the case for the final pH (i.e., the PRE reached 71.6 ± 2.5 % at pH 10.5 and 91.2 ± 1.3 % at pH 11.5) (Fig. 5). Alternatively, when considering 4x water dilution, as far as the CD increased (so a shorter electrolysis time was applied), a decrease in the PRE was observed. Thus, the PRE decreased from 61.2 ± 8.3 % to 18.0 ± 11.5 % when raising the CD from 0.4 to 1.2 A m^−2^ and considering as final pH 10.5, and from 89.5 ± 0.4 % to 71.1 ± 2.8 % when final pH was 11.5. In line with Company et al. [29], higher PREs were reached at pH 11.5 than at pH 10.5. Owing to the high working pH, the removal percentages for undiluted water reached high levels when compared to other EMP processes operated at low CD (Table 2). The sEC_P_ was affected by the effluent strength and calculated in the range from 14.1 ± 3.1 kWh kg^−1^ P (dilution factor 1x, final pH 10.5, and CD 0.4 A m^−2^) to 81.0 ± 16.2 kWh kg^−1^ P (dilution factor 4x, final pH 11.5, and CD 1.2 A m^−2^). According to Fig. 4, the higher sEC_P_ was associated with the lower concentration of P in the wastewater, as already reported by Lei et al. [20,35]. A comparison of the sEC_P_ values obtained in this research with others found by other authors is presented in Table 2. Interesting values between 4.4 and 26.4 kWh/kg P were reported by Ref. [28] when working with low-strength wastewater at CD in the range 0.04–0.2 A/m^2^.

**Table 2:** Summary of references achieving phosphorus recovery by electrochemical mediated precipitation (EMP) processes operated with low current density.

CD (A m^−2^)	Anode	Membrane	Cathode	Final product	Concentration (mg P L^−1^)	pH_t0_ (−)	pH_tf_ (−)	PRE (%)	sEC_P_ (kWh kg^−1^ P)	Ref.
0.4–1.2	Ti–MMO	CEM	Stainless-steel	CaP MgP	48	8.5	10.5	69–74	14–20	This work
							11.5	90–93	26–37	
					12		10.5	18–61	39–78	
							11.5	71–88	52–81	
0.4–1.3	Graphite	(M)CEM	Graphite	CaP	12	7	10	83–90	62–88	[24]
0.04–0.2	Pt–Ti	–	Pt–Ti Graphite felt	CaP	8–19	7.8	4	≤70	4–26	[28]
1.4	Pt coated Ti	–	Pt	CaP	7	–	–	44	110	[20]
1.9	Ru–Ir coated Ti	–	Stainless-steel	CaP	789	4.5	5	40	27	[35]

CaP, calcium phosphate; CD, current density; CEM, cation exchange membrane; MgP, magnesium phosphate; PRE, phosphorus removal efficiency; sEC_P_, specific energy consumption in relation to P removal from the liquid phase.

Together with the concentration of PO_4_^3-^-P, the concentration of Ca^2+^ and Mg^2+^ in the catholyte also decreased (Fig. 5), especially in the case of testing the undiluted effluent. However, the removed Ca/P and Mg/P molar ratios were usually lower than 1.00–1.67, which is the typical range for calcium and magnesium phosphates [6]. This performance could be explained by the kind of membrane used (i.e., CEM), which allows cations to cross from the anodic to the cathodic compartment, thus affecting the concentration.

#### 3.1.2 Mineral phase formed in the cathodic compartment

The turbidity of the catholyte increased throughout the electrochemical tests due to the formation of small crystallization nuclei, but no visible precipitate was found at the bottom of the cathodic compartment by the end of the experiments. Kappel et al. [27] already stated that a high pH in the catholyte causes immediate crystallization of the phosphate, which remains suspended in the liquid. After crystallization, a final separation by centrifugation, settling or filtration allows the final recovery of phosphate compounds.

**Fig. 6:**
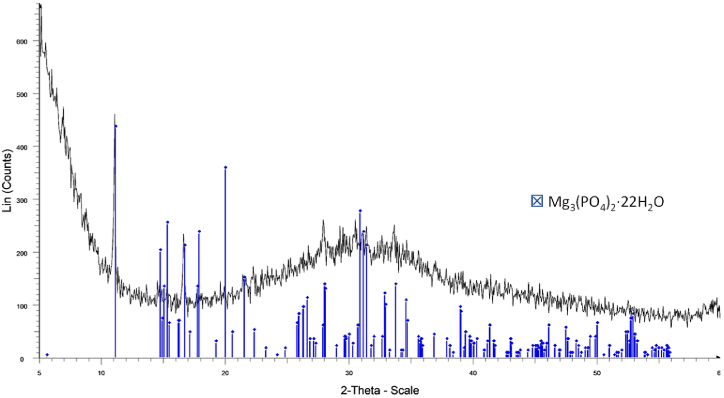
XRD pattern for the solids collected from the catholyte by the end of a batch test (undiluted effluent, 1.2 A m^−2^, and pH 11.5). The liquid was filtered, and the retained solids were dried at room temperature and ground before analysis.

The supersaturation conditions in the catholyte were assessed through the calculation of the SI in order to identify the crystallization of possible mineral phases (Table S1). Beyond calcium phosphate (CaP) -which tends to form first (basically as an amorphous phase [42])-, when considering magnesium phosphates (MgP), the highest SI was reached by bobierrite (Mg_3_(PO_4_)_2_·8H_2_O) followed by cattiite (Mg_3_(PO_4_)_2_·22H_2_O), which forms faster and switches from unstable to stable state in air when dried at room temperature [39]. Potential formation of Ca- and Mg-carbonates and brucite was also envisaged. Both elements, Mg and P are considered as critical raw materials for the EU [43]. The XRD analysis of the solids formed in the catholyte when working with undiluted denitrified effluent at 1.2 A m^−2^ revealed that cattiite may occur as a crystalline phase but that other amorphous phases were formed in a significant proportion (Fig. 6). According to the ICP-OES analysis (Table 3), the precipitated salt showed a prevalent content in Mg, followed by Ca and P (11.0 %, 6.9 %, and 5.4 %, respectively), which is equivalent to Ca/P and Mg/P molar ratios of 1.3 and 2.0, respectively (suggesting carbonates or brucite formation). These results are consistent with Company et al. [29], who already pointed out that when raising the pH of the swine denitrified effluent, if ammonium is not available, cattiite may be the prevalent crystal formed rather than other salts belonging to the family of the struvites. In this regard, an experiment using NaOH to raise the pH (11.0) of the denitrified effluent clearly showed the formation of cattiite through a faster process leading to larger crystals than in the electrochemical system. The composition of the mineral phase in such case was quite different (10.6 % P, 9.2 % Mg, and 2.2 % Ca). A multinutrient product is commonly obtained when addressing phosphate precipitation from complex wastewaters such as denitrified livestock effluents. For an enhanced fertilization with this kind of precipitates it is encouraged to consider the real composition of the precipitated product rather than the theoretical formula of a particular crystal.

**Table 3:** Composition analyzed by ICP-OES of the precipitate collected from the catholyte when working with undiluted effluent at 1.2 A m^−2^. Results are expressed in percentage on dry weight basis (solids dried at room temperature).

Element	Catholyte
P	5.4
Ca	6.9
Mg	11.0
K	1.0
Na	0.28

#### 3.1.3 Chlorine production in the anodic compartment

In an electrolysis process, hydrogen is produced at the cathode and oxygen at the anode (Eqs. (1), (2))). Moreover, in presence of chloride in the media, oxygen-evolving anodes can oxidize chloride into hypochlorite (ClO^−^), which is in chemical equilibrium with chlorine gas (Eq. (3); the potential of the anode must reach a minimum of about +1.4 V vs. SHE [44]). Both hypochlorite and chlorine gas are strong oxidizing agents that were commonly produced by the mercury-based chlor-alkali process until the EU Regulation 2017/852 banned the use of mercury in such process. Since then, the use of electrochemical methods for their production has shown a growing interest [45,46]. To evaluate chlorine formation as a side-product in the anodic compartment, chlorine concentration was measured in the anolyte at the end of each test. Considering treatment of undiluted effluent, the CD boosted the chlorine production, resulting in 0.6 ± 0.02 and 1.2 ± 0.03 mg Cl_2_ L^−1^ for 0.4 and 0.8 A m^−2^, respectively, and reaching the maximum value of 3.0 ± 0.3 mg Cl_2_ L^−1^ at 1.2 A m^−2^. Nonetheless, the chlorine concentration measurement was likely to be limited since the Schott bottles in which the anolyte was recirculated (Fig. 1) were not sealed. In the context of a farm, hypochlorite/chlorine gas could be recovered downstream the electrochemical system and used for disinfection tasks on-site. Despite of the strong reactivity, it is not expected that these compounds modify the characteristics of the P products formed because *(i)* P is present in the form of (ortho)phosphate and it cannot be further oxidized and *(ii)* precipitated phosphate salts are formed at the cathode and chlorine is formed at the anode. Both compartments are separated by an ion exchange membrane. The preliminary results of this work concerning chlorine production suggest that the accumulation of this product could be feasible when treating denitrified swine effluent, but the electrochemical system must be optimized.

### 3.2 Catholyte neutralization in the anodic compartment

According to the results reported above, pH 11.5 allows for intensifying P crystallization in the cathodic compartment. However, effluent water cannot be discharged neither used for irrigation, at such high pH value. High-pH water can negatively affect nutrient availability in soil by reducing micronutrient solubility but, on the contrary, low-pH recycled water can lead to an increased metals mobility, contributing to the contamination of the water bodies [47]. Thus, catholyte neutralization within a specific pH range is a critical factor in view of subsequent water reuse for irrigation. As an alternative to acid dosage to correct the pH, catholyte neutralization was evaluated at the anodic compartment.

**Fig. 7:**
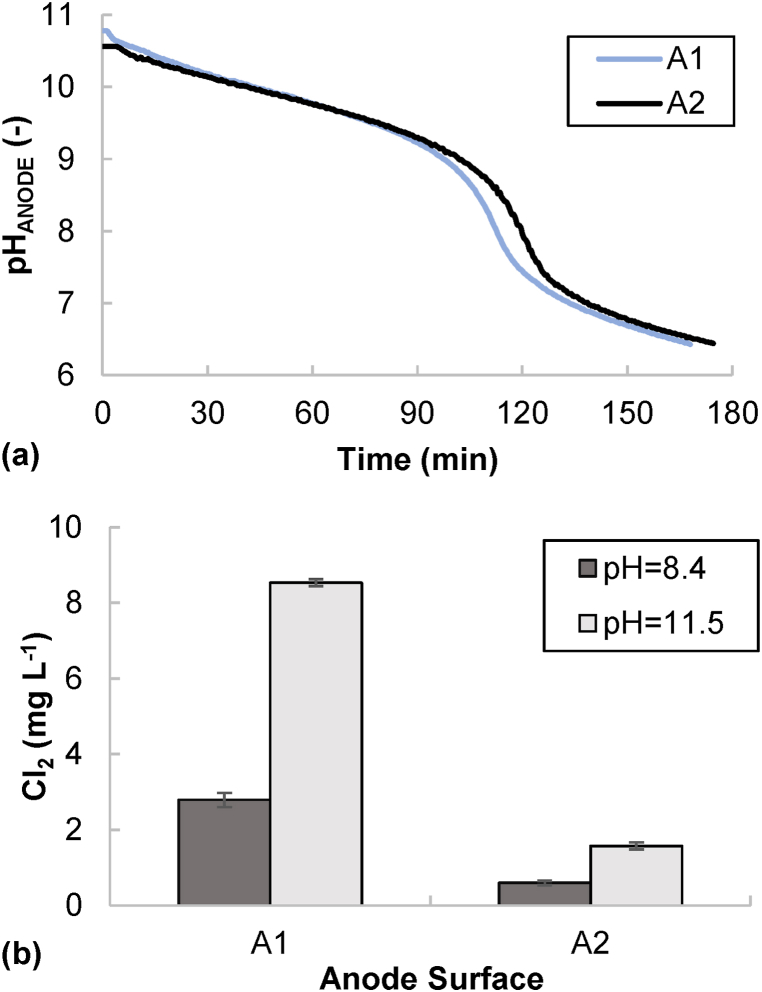
Catholyte neutralization batch tests considering different anode areas (A1: 148 cm^2^; A2: 444 cm^2^). (a) pH against time profile. (b) Final chlorine concentrations considering different initial pH of the anolyte.

The PRE was not affected by the different composition of the anolyte (fresh denitrified effluent vs. final catholyte from a previous batch test) nor by the different anode surface tested, reaching values of ca. 90 % (final pH: 11.5, CD: 1.2 A m^−2^). The increase in the anode surface did not influence the pH profiles either, which typically presented a steeper slope change in the range 9.5–7.0 (Fig. 7a). When using the catholyte from a previous test as the anolyte (pH_0_ ∼11.0), its pH reached the neutrality (6.4 ± 0.1) at the end of the test. This value is acceptable for agricultural applications (i.e., common required pH values in agricultural water reuse regulations are in the range from 6.0 to 9.0 [47]). When using fresh denitrified effluent (pH_0_ ∼8.5), the anolyte pH decreased to 5.3 ± 0.1. The final EC of the anolyte when using the catholyte from a previous test was measured as 5.9 ± 0.2 dS m^−1^, which was slightly below the value for the fresh denitrified effluent (Table 1). These are promising results for the operation of the electrochemical system under continuous-flow mode, since the final catholyte could be directly neutralized in the anodic compartment, thus avoiding the dosage of chemical reagents, and reducing the treatment cost.

The anode surface played a role in the chlorine formation. The measured concentration in the anolyte (if considering the catholyte from a previous test) was 8.6 ± 0.1 and 1.5 ± 0.4 mg Cl_2_ L^−1^ when considering 148 and 444 cm^2^ as the anode surface, respectively. Thus, the larger the anodic surface, the lower the concentration of chlorine in the anolyte by the end of the test. The chlorine formation also depended on the anolyte used. The use of the catholyte from the previous batch tests (pH 11.5) showed a higher chlorine production than the use of the fresh denitrified effluent, which could be related with the higher pH of the catholyte. At higher pHs, the chlorine equilibrium is moved to hypochlorite (OCl^−^), which limit the losses related to Cl_2_ volatilization. Thus, the higher the initial pH value, the higher the measured concentration of chlorine (Fig. 7b). In conclusion, catholyte neutralization through the anodic compartment was proven as feasible. Moreover, chlorine production was favored by using spent catholyte as influent owing to its high pH.

### 3.3 EIS and membrane analyses

#### 3.3.1 EIS tests

**Fig. 8:**
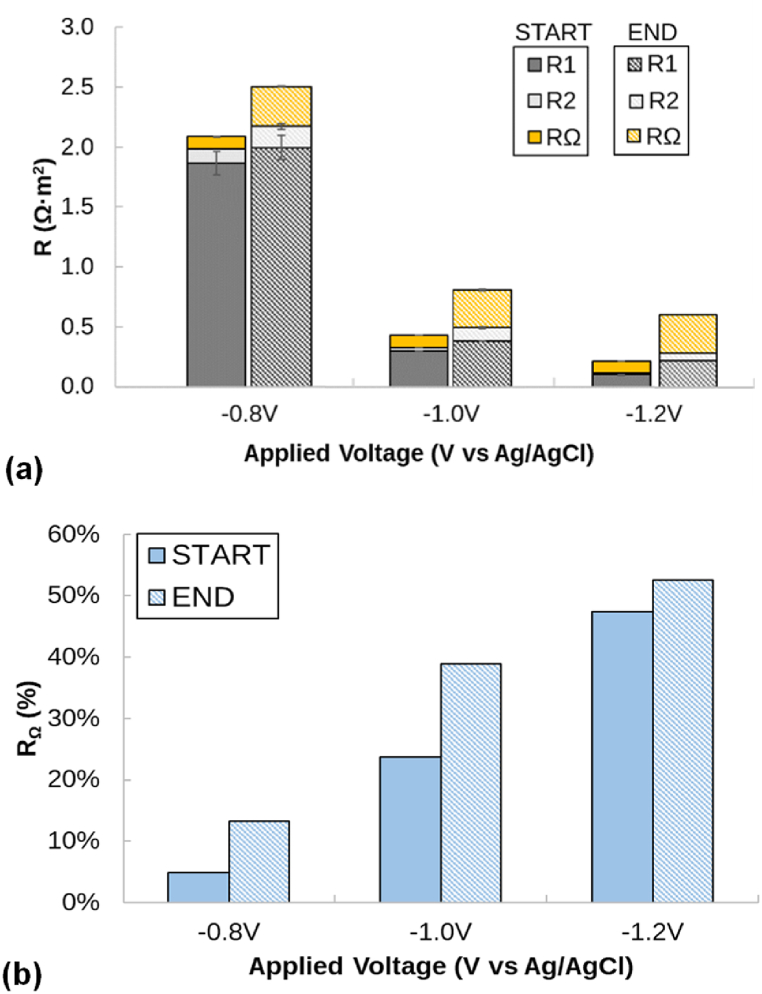
Resistances calculated by fitting the EIS spectra to equivalent electrical circuit models at different voltages through the three-electrode configuration. (a) Cathode-related resistances (R_1_ and R_2_) and ohmic resistance (R_Ω_) measured in Ω·m^2^. (b) R_Ω_ measured as a percentage of the total internal resistance (R_1_, R_2_ and R_Ω_). Start and End labels identify the initial and final conditions before and after performing the precipitation tests.

The mineral deposition on the electrode surface is expected to decrease the effective surface area of the electrode, and thus, to increase the internal resistance of the system. EIS was applied to evaluate this issue. EIS is a fast and non-destructive technique offering kinetics and mechanistic data that could be used to study the performance of the electrochemical systems and, particularly, the overall resistance components [48]. In this study, two EIS runs were performed to characterize the system's electrochemical performance prior to and after conducting the precipitation tests. The EIS spectra and the considered equivalent circuits for the current study are shown elsewhere (Fig. S2). The two-electrode test (in OCV mode) allow characterization of the total internal resistance of the electrochemical system, resulting in 1.20 ± 0.06 Ω m^2^. From this test, the resistance related to the flow of ions between electrodes (i.e., the ohmic resistance (R_Ω_) -which is the value resulting from the EIS spectra intersection with the X-axis multiplied by the cathode surface-) was estimated as 0.56 Ω m^2^, thus contributing to 47 % of the total resistance. Concerning the three-electrode test, three main resistances (R_1_, R_2_ and R_Ω_) were identified (Fig. 8). R_1_ and R_2_ were related to the working electrode (i.e., the cathode) and showed large variability depending on the applied voltage –which is attributable to electron transport processes such as those happening at the electrodes [49]. The ohmic resistance (R_Ω_) increased from the start to the end of the test. When the system was operated at a higher cell voltage, the cathodic resistance decreased due to the activation of H_2_ evolution reaction, but the ohmic resistance increased due to the precipitation over the electrode (i.e., blocking of active sites, formation of insulating layers) and reduction of the ion conductivity due to the loss of ions. Taking it all together, the relevance of the R_Ω_ with respect to the total cathodic compartment resistance increased as far as the cell voltage was increased (Fig. 8).

Nonetheless, the ohmic resistance in the electrochemical system after carrying out all the precipitation tests was 0.79 (two-electrode test) and 0.23 Ω m^2^ (three-electrode test) higher than the initial resistance. Specifically, when 1.2 A m^−2^ was applied in the precipitation test with undiluted effluent, a 0.08 Ω m^2^ increase was detected with the two-electrode test. These results suggest a limited mineral deposition on the cathode and on the membrane surface due to the pH increase. As an example, Zhu et al. [50] observed 3-fold higher ohmic resistances (815 and 848 Ω m^2^) when applying 3-fold higher currents (600 and 2000 A m^−2^) in new Proton Exchange Membrane Fuel Cells.

#### 3.3.2 Membrane analyses

**Fig. 9:**
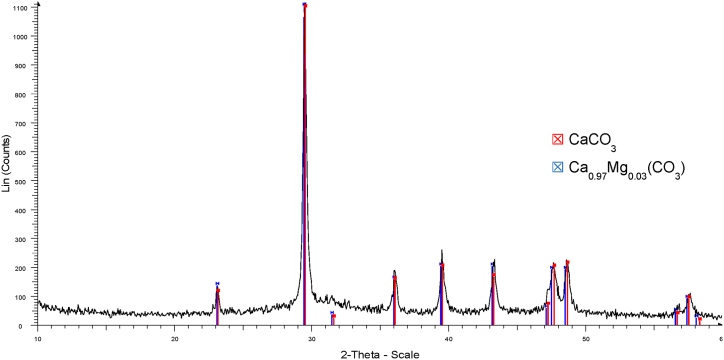
XRD pattern of the solids collected from the CEM at the end of the precipitation tests (once the electrochemical system had been disassembled).

**Fig. 10:**
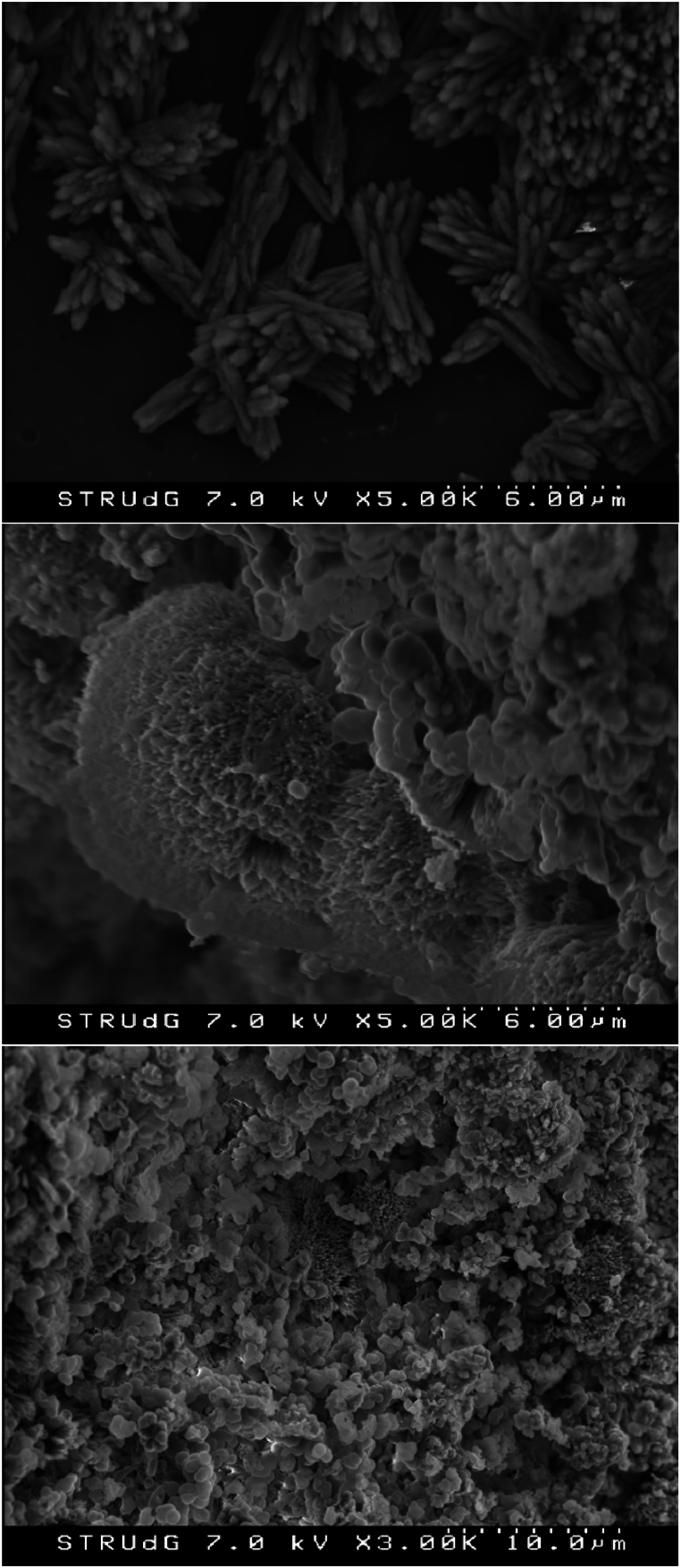
SEM images of the solids collected from the CEM at the end of the precipitation tests (once the electrochemical system had been disassembled).

At the end of the precipitation tests the electrochemical system was disassembled, which revealed scaling on the membrane surface at the cathode side. In contrast to the bioelectrochemical systems, where biofouling tends to occur on the membrane [51], in this case, the XRD analysis of the precipitate attached to the membrane (Fig. 9) revealed that carbonate compounds such as calcite (CaCO_3_) and magnesium calcite (Ca_x_Mg_y_(CO_3_)) were deposited on the membrane surface. This was likely because of the high pH-values obtained owing to the OH^−^ produced, which led to an increased concentration of the carbonate ion (CO_3_^2−^) that promoted its precipitation [42,52]. Such deposition could be the main reason for the increase of the ohmic resistance observed in the two-electrode EIS test (i.e., while the ohmic resistance detected with the three-electrode test is representative of the cathode compartment, the resistance in the two-electrode test is influenced by the overall system also including the membrane). SEM images showed the morphologies of the solids formed (Fig. 10). The composition of the solids deposited on the membrane at the end of the experimental period was analyzed by ICP-OES (Table 4). Concerning the membrane deposits, results were in line with those of XRD, since Ca was the main element detected (29.6 % on dry weight basis). The elements P and Mg were also present, but in a more limited percentage (4.7 % and 2.3 %, respectively). Thus, the presence of Ca and inorganic carbon in the denitrified effluent strongly affected the characteristics of the membrane deposits but also induced an interference on the formation of MgP in the cathodic compartment.

**Table 4:** Composition analyzed by ICP-OES of the deposits collected from the surface of the cationic exchange membrane (CEM) at the end of precipitation experiments. Results are expressed in percentage on dry weight basis (solids dried at room temperature).

Element	Membrane
P	4.7
Ca	29.6
Mg	2.3
K	0.09
Na	0.12

### 3.4 Operational costs

The energy cost related to the operation of the electrochemical system when treating undiluted denitrified effluent (pH 11.5) was estimated to be between 0.20 ± 0.02 € m^−3^ (0.4 A m^−2^) to 0.31 ± 0.03 € m^−3^ (1.2 A m^−2^). On the other hand, the cost of dosing NaOH to raise the pH to 11.5 in a conventional crystallizer was estimated as 0.46 € m^−3^ and the cost of dosing H_2_SO_4_ for neutralization purposes was estimated as 0.25 € m^−3^. Thus, the total cost for the chemically mediated pathway increases to 0.71 € m^−3^. In terms of precipitated P, the operational cost when considering the addition of chemical reagents (PRE of 90 % at pH 11.5), was calculated as 16.5 € kg^−1^ P. This cost is higher than the cost calculated for the energy consumption in the electrochemical system (CD ≤ 1.2 A m^−2^ and final pH 11.5), which is in the range from 5.1 ± 0.7 € kg^−1^ P to 8.4 ± 2.1 € kg^−1^ P. In a more detailed economic assessment, the cost of the electrochemical process should also include charges for the operation of the pumping system and the replacement of the electrodes and membrane. Nonetheless, these preliminary results show the EMP process at low CD as an attractive alternative to chemical precipitation. The options of recovering chlorine as a valuable side-product have not been considered, neither. Yet, the estimated costs for the EMP process are still higher than those referred to for the mined P (1.5–2.0 € kg^−1^ P) [53,54], so the optimization of the electrochemical process is necessary to make the process economically viable.

## 4 Conclusions

Feasibility for chemical-free P precipitation from a denitrified swine effluent was successfully demonstrated at pH 10.5–11.5 using a two-chamber batch electrochemical reactor equipped with a CEM and operating at low CD (0.4–1.2 A m^−2^). The main conclusions reached are as follows.

• For the undiluted effluent (48 mg P L^−1^), once the targeted pH was defined, the PRR increased according to the CD applied. Maximum PRR of 33.4 mmol P (L·d^−1^) with PRE ≥90 % was reached when targeting pH 11.5 and applying 1.2 A m^−2^ (41.8 kWh kg^−1^ P). The sEC_P_ behaved inversely to the effluent strength, ranging (at pH 11.5) from 25.8 kWh kg^−1^ P (0.4 A m^−2^, 1x dilution) to 81.0 kWh kg^−1^ P (1.2 A m^−2^, 4x dilution). According to this, higher initial concentrations of P favored energy savings. Limited deposition of solids occurred during the tests and the solids formed mostly remained in suspension.
• Neutralization of the catholyte in the anodic compartment was demonstrated, achieving a final pH of 6.4 ± 0.1 (which is a suitable value for irrigation).
• Chlorine production in the anode was favored by a high initial pH of the catholyte and a small anode surface. These findings represent a new opportunity for the recovery and onsite use of this side-product.
• The EIS tests confirmed that the deposition of solids into the system throughout the experimental period was limited, only contributing to a slight increase of the ohmic resistance (quantified as 0.79 Ω m^2^ in the two-electrode configuration test). Membrane analysis revealed precipitation of carbonate compounds.
• The electrochemical system was shown as a promising alternative to NaOH dosage for pH adjustment when targeting P recovery from denitrified effluents, eventually allowing for economical savings.
• Future investigations should involve testing the electrochemical system running in continuous-flow mode, evaluating different configurations to enable solids recovery, and exploring chlorine recovery. Such endeavors could advance the practical application of this method as a sustainable wastewater treatment process to recover valuable products.

## Data availability statement

Data was not deposited into a publicly available repository. Data will be made available on request.

## CRediT authorship contribution statement

**Emma Dessì:** Writing – original draft, Visualization, Validation, Methodology, Investigation, Data curation, Conceptualization. **Emma Company:** Writing – review & editing, Validation, Methodology, Investigation, Data curation, Conceptualization. **Narcís Pous:** Writing – review & editing, Resources, Methodology, Conceptualization. **Stefano Milia:** Writing – review & editing, Supervision. **Jesús Colprim:** Writing – review & editing, Validation, Supervision, Resources, Methodology, Funding acquisition, Conceptualization. **Albert Magrí:** Writing – review & editing, Writing – original draft, Visualization, Validation, Supervision, Methodology, Funding acquisition, Data curation, Conceptualization.

## Declaration of competing interest

The authors declare the following financial interests/personal relationships which may be considered as potential competing interests:

Albert Magrí reports financial support was partially provided by 10.13039/501100002809Government of Catalonia. If there are other authors, they declare that they have no known competing financial interests or personal relationships that could have appeared to influence the work reported in this paper.
