# Supplementary material for: Reagent-free phosphorus precipitation from a denitrified swine effluent in a batch electrochemical system
Source: Heliyon. 2024 Aug 23;10(17):e36766. doi: 10.1016/j.heliyon.2024.e36766 (PMC11387353; doi:10.1016/j.heliyon.2024.e36766)
Supplement: Multimedia component 1 [file mmc1.docx]

**SUPPLEMENTARY MATERIAL**

**Reagent-free phosphorus precipitation from a denitrified swine effluent in a batch electrochemical system**

Emma Dessì^a,b,1^, Emma Company^a,1^, Narcís Pous^a^, Stefano Milia^c^, Jesús Colprim^a^, Albert Magrí^a,*^

^a^ Laboratory of Chemical and Environmental Engineering (LEQUIA), Institute of the Environment, University of Girona, Girona, Spain.

^b^ University of Cagliari – Department of Civil-Environmental Engineering and Architecture (DICAAR), Cagliari, Italy.

^c^ National Research Council – Institute of Environmental Geology and Geoengineering (CNR-IGAG), Cagliari, Italy.

^1^ Both authors contributed equally to this work.

* Corresponding author.

**Fig. S1.** pH against time profiles in the catholyte of the batch electrochemical system for the (a) 1x ‒undiluted‒ and (b) 4x diluted denitrified effluent.

**Table S1**

Saturation indexes (SIs) for possible mineral phases (SI>0) formed in the catholyte of the electrochemical system as a function of the pH value. Calculations were made using Visual MINTEQ and based on ionic concentrations in the denitrified swine effluent (Table 1).

|  | **pH** | | | |
| --- | --- | --- | --- | --- |
|  | **10.0** | **10.5** | **11.0** | **11.5** |
| **Mineral phase** | **SI (-)** | | | |
| Hydroxyapatite (Ca₁₀(PO₄)₆(OH)₂) | 16.16 | 16.64 | 17.02 | 17.32 |
| Calcium phosphate (Ca_3_(PO_4_)_2_) (beta) | 4.85 | 4.91 | 4.90 | 4.83 |
| Bobierrite (Mg_3_(PO_4_)_2_·8H_2_O) | 4.05 | 4.56 | 5.01 | 5.26 |
| Cattiite (Mg_3_(PO_4_)_2_·22H_2_O) | 1.94 | 2.44 | 2.89 | 3.15 |
| Magnesite (MgCO_3_) | 1.84 | 1.91 | 1.92 | 1.87 |
| Calcite (CaCO₃) | 1.88 | 1.81 | 1.66 | 1.51 |
| Dolomite (CaMg(CO_3_)_2_) (disordered) | 4.32 | 4.32 | 4.18 | 3.98 |
| Huntite (CaMg_3_(CO_3_)_4_) | 6.51 | 6.65 | 6.52 | 6.23 |
| K-Struvite (MgKPO_4_·6H_2_O) | 1.53 | 1.83 | 2.08 | 2.25 |
| Na-Struvite (MgNaPO_4_·7H_2_O) | 0.76 | 1.05 | 1.30 | 1.46 |
| Brucite (Mg(OH)_2_) | -0.25 | 0.66 | 1.59 | 2.51 |

**Fig. S2.** Three-electrode electrochemical impedance spectroscopy (EIS) spectra at different voltages (values for the potential -0.8 V must be read in the principal axes whereas values for the potentials -1.0 and -1.2 V must be read in the secondary axes) corresponding to (a) the beginning of the experimental period ‒once assembled the electrochemical system but before performing any experiment‒ and (b) the end of the experimental period ‒once performed all the experiments‒. Two-electrode EIS spectra corresponding to (c) the initial and final condition described above. Dots represent experimental values and dashed lines represent fittings achieved. Equivalent electric circuits model (ECM) used for fitting the EIS spectra in (d) the two-electrode tests and (e) the three-electrode tests. The circuit components are resistances (R), constant phase elements (Q) and a Warburg element (W). The EIS spectra intersection with the x-axis identifies the ohmic resistance (R_3_), which usually is expressed considering the cathode surface (Ω·m^2^).
